# Salivary Proteome Profile of Xerostomic Patients Reveals Pathway Dysregulation Related to Neurodegenerative Diseases: A Pilot Study

**DOI:** 10.3390/ijms26157037

**Published:** 2025-07-22

**Authors:** Abhijeet A. Henry, Micaela F. Beckman, Thomas S. Fry, Michael T. Brennan, Farah Bahrani Mougeot, Jean-Luc C. Mougeot

**Affiliations:** 1 Translational Research Laboratories, Cannon Research Center, Atrium Health Carolinas Medical Center, Charlotte, NC 28203, USA; abhijeet.henry@atriumhealth.org (A.A.H.); micaela.beckman@atriumhealth.org (M.F.B.); 2 Department of Bioinformatics and Genomics, College of Computing and Informatics, Charlotte, NC 28223, USA; 3 Department of Oral Medicine/Oral and Maxillofacial Surgery, Wake Forest University School of Medicine, Atrium Health Carolinas Medical Center, Charlotte, NC 28203, USA; tom.fry@dhha.org (T.S.F.); mike.brennan@atriumhealth.org (M.T.B.); 4 Department of Dentistry, Denver Health Hospital Authority, Denver, CO 80204, USA

**Keywords:** saliva, xerostomia, dry mouth, salivary hypofunction, proteomics, neurodegenerative diseases

## Abstract

Xerostomia, the subjective complaint of a dry mouth, is frequently associated with salivary flow reduction and/or salivary gland hypofunction. This condition significantly impacts an individual’s quality of life and oral health, including difficulties in speaking, chewing, and swallowing. Xerostomia may be caused by autoimmune diseases, xerogenic medications, and radiation therapy. Our objective was to identify differentially expressed proteins in the saliva of patients with medication and autoimmune disease-associated xerostomia compared to non-xerostomic control subjects. Two groups of individuals (N = 45 total) were recruited: non-xerostomic subjects (NX-group; *n* = 18) and xerostomic patients (XP-group; *n* = 27). Dried saliva spot samples were collected from major salivary glands, i.e., parotid (left and right) and submandibular glands. Proteomic analysis was performed by deep nanoLC-MS/MS. Differential protein expression in the XP-group relative to the NX-group was determined by the Mann–Whitney U-test with FDR Benjamini–Hochberg correction (*p*_adj_ < 0.05). The Search Tool for Recurring Instances of Neighboring Genes (STRING_v12.0_) was used to generate interaction networks and perform pathway analysis. A total of 1407 proteins were detected. Of these, 86 from the left parotid gland, 112 from the right parotid gland, and 73 from the submandibular gland were differentially expressed proteins (DEPs). Using STRING analysis, we identified, for the first time, several neurodegenerative disease-associated networks, primarily involving the downregulation of the 20S proteasome core complex and glyoxalase proteins across salivary glands. In this study, we determined neuronal dysregulation and impaired methylglyoxal (MGO) detoxification, possibly through reduced protein expression of glyoxalase Parkinson’s Disease (PD) Protein 7 (encoded by the *PARK7* gene) in major salivary glands of xerostomic patients. Indeed, impaired MGO detoxification has been previously shown to cause salivary gland dysfunction in a mouse model of type 2 diabetes. Based on other DEPs associated with neurodegenerative disorders, our results also suggest a possible deficiency in the parasympathetic nervous system innervation of salivary glands, warranting further investigation.

## 1. Introduction

Saliva is secreted by parotid, submandibular, sublingual glands, and minor salivary glands and plays a crucial role in oral health by lubricating the oral mucosa and maintaining mucosal integrity. Saliva is composed of water and organic and non-organic substances such as enzymes, hormones, antibodies, and growth factors [1]. Xerostomia, commonly known as dry mouth, occurs when these glands fail to produce adequate saliva volume and/or quality. Xerostomia symptoms include difficulty speaking, chewing, and swallowing and an increased risk of dental caries and oral infections; therefore, they may significantly impact an individual’s quality of life. Xerostomia may also be associated with a noticeable reduction in salivary flow [2]. Xerostomia affects approximately 30% of the world population aged over 65 [3]. The condition predominantly affects postmenopausal women and cancer patients undergoing radiation therapy [4,5].

Xerostomia development involves multiple factors that contribute to the pathology of the condition. Reductions in estrogen and progesterone during menopause have been associated with the development of xerostomia, as the oral mucosal cells and salivary glands are found to have receptors for sex hormones [6]. Autoimmune diseases such as Sjögren’s disease significantly contribute to the development of xerostomia due to chronic inflammation and lymphocytic infiltration of lacrimal and salivary glands [7]. However, polypharmacy (the prescription of multiple medications) is regarded as the most common cause of xerostomia, explaining the association between the elderly population and dry mouth. From 2005 to 2011, in the United States, more than 75% of people over the age of 65 took at least one medication prescription that may affect salivary function [8]. Other causes include uncontrolled diabetes, sialolithiasis, amyloidosis, sarcoidosis, and various rheumatic diseases [1].

Patients experiencing xerostomia are often prescribed medications such as pilocarpine and cevimeline, used to stimulate residual glandular function, whereas other treatments include hormone replacement therapy (HRT), salivary substitutes, and salivary pacemakers [1]. HRT, involving the use of estrogen and progesterone, has shown some effectiveness in alleviating menopausal symptoms, including xerostomia, by increasing salivary flow rates [9]. Salivary substitutes, available in liquid, spray, or gel forms, provide temporary relief by mimicking the lubricating function of natural saliva [10]. Salivary pacemakers offer a more innovative solution by using neuro-electrostimulation to enhance saliva production [11]. Previous research has explored potential therapeutic agents, such as cholinesterase inhibitors, malic acid, and anethole trione, which have shown promise in clinical studies for their ability to stimulate residual glandular function [12,13,14]. Other investigational approaches include monoclonal antibodies like rituximab and epratuzumab, as well as coenzyme Q10 that targets oxidative stress mechanisms [15,16]. Gene therapy, particularly those focusing on the expression of aquaporin genes, such as Aquaporin 1 (AQP1) and Aquaporin 5 (AQP5), has shown potential in improving salivary flow and offers a novel avenue for restoring salivary gland function [17,18].

Saliva has emerged as a valuable non-invasive proteomic diagnostic tool in personalized medicine as it is rich in both lubricant proteins and components of the innate immunity system, offering antimicrobial activity from microbial proteases [19]. Additionally, proteins from blood can enter saliva through inflamed gingivae and microinjuries, with 27% of the saliva proteome shared with the blood plasma proteome [20,21]. Advances in ‘-omic’ research technologies have further enhanced the utility of saliva for diagnosing different pathologies, with the biomarkers being detected for various infectious, oral, and neurological diseases, as well as several cancer types [19]. Most studies incorrectly view saliva as a homogeneous body fluid, but saliva is not a solitary fluid and is a complex mixture of secretions from three major glands: the parotid, submandibular, and sublingual glands. Each gland secretes proteins with unique profiles based on their differing ratios of serous and mucous acinar cells, contributing to saliva’s complex proteome. The parotid gland is primarily responsible for serous secretions abundant in salivary alpha-amylase for carbohydrate digestion, and the submandibular–sublingual glands produce mucous-rich saliva for lubrication, protection, and maintenance of the oral cavity [22]. The growing potential of salivary proteomics, notably through the identification of differentially expressed proteins between healthy and diseased individuals, offers significant opportunities to understand various pathological mechanisms in Xerostomia. However, studies have observed discrepancies among published proteomic datasets, due to possible differences in collection methods, sample integrity, storage conditions, and analytical approaches, hampering the use of saliva as a reliable research fluid and highlighting the need for standardization [23]. Along with distinct protein secretion profiles found among salivary glands, these findings support the need for a gland-specific approach to proteomic analysis employed in our experiment [23].

In this study, we aimed to explore xerostomia at the molecular level by identifying differentially expressed proteins (DEPs) in dried saliva spot samples from the left parotid, right parotid, and submandibular glands of patients with medication and autoimmune disease-associated xerostomia compared to non-xerostomic subjects. We hypothesized that we would identify possible important molecular mechanisms underlying xerostomia, potentially aiding in the development of personalized therapies in the future.

## 2. Results

**Figure 1:**
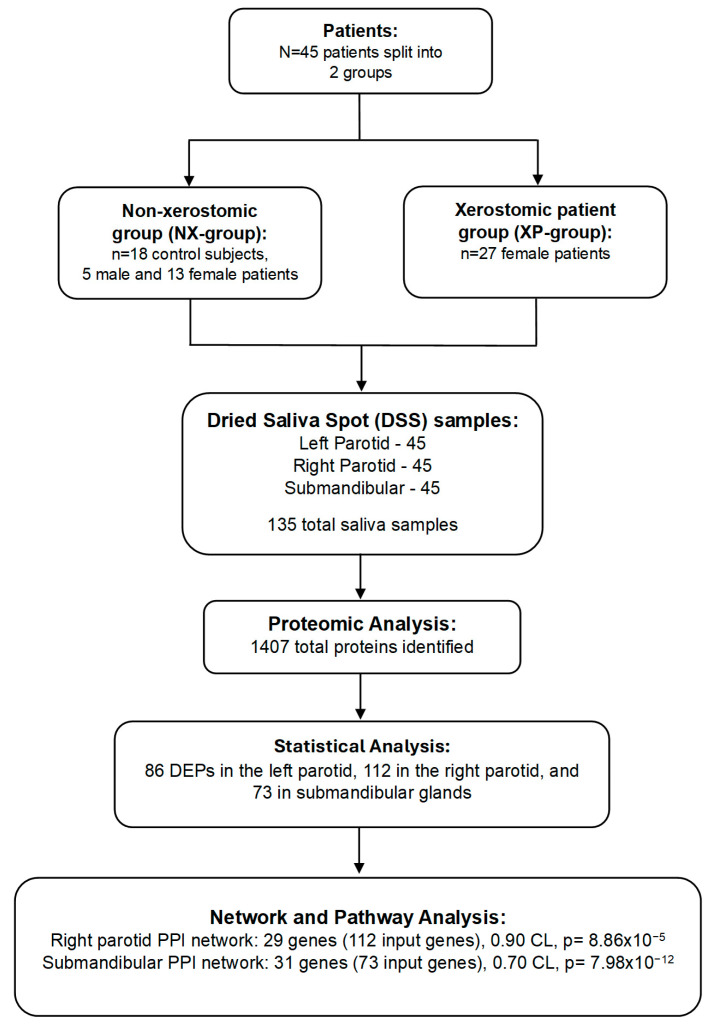
Experimental design flowchart. **Legend.** Patients (N = 45) were divided into 2 groups: non-xerostomic control subjects (NX-group) consisting of 5 males and 13 females and xerostomic patients (XP-group) consisting of 27 females. Dried saliva spot samples were collected from the left parotid, right parotid, and submandibular salivary glands, for a total of 135 saliva samples. Proteomic analysis identified a total of 1407 proteins across all three salivary gland sites, followed by the identification of differentially expressed proteins (DEPs) for each salivary gland. Statistical analysis revealed 86 DEPs in the left parotid, 112 in the right parotid, and 73 in the submandibular glands, which were subsequently used as input genes to create protein–protein interaction (PPI) networks from The Search Tool for Recurring Instances of Neighboring Genes (STRING_v12.0_) for each gland site. The right parotid network resulted in 29 output genes with a confidence level of 0.90 (*p* = 8.86 × 10^−5^) and the submandibular network resulted in 31 output genes with a confidence level of 0.70 (*p* = 7.98 × 10^−12^). The Kyoto Encyclopedia of Genes and Genomes (KEGG_v112.1_) was used for pathway analysis.

The overall methodology and results are presented in Figure 1.

Patient demographics. Demographics for non-xerostomic subjects (NX-group) and xerostomic patients (XP-group) are presented in Table 1. Reported comorbidities and medications by participants in each group are presented in Supplemental Table S1.

**Figure 2:**
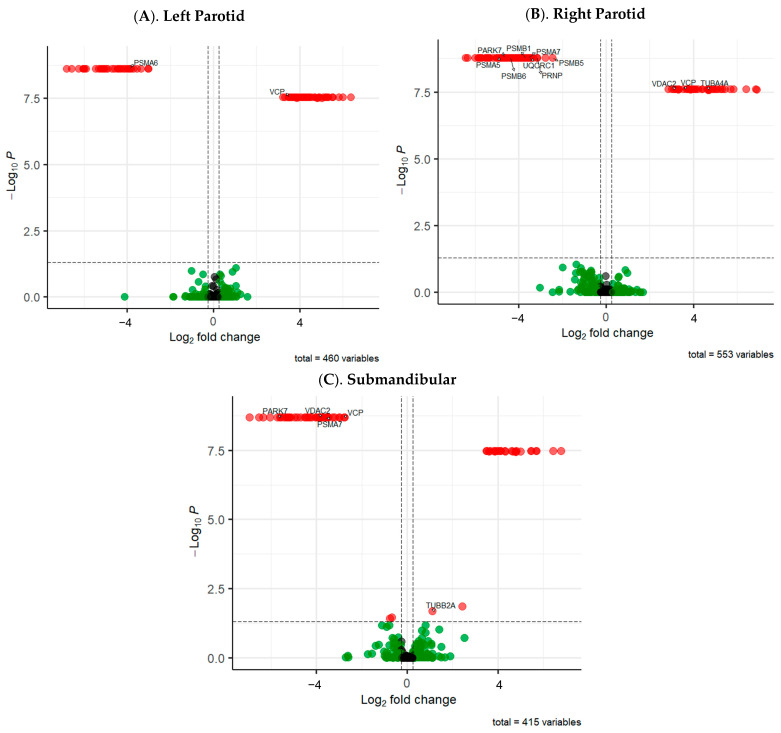
Volcano plots for control vs. xerostomia groups. **Legend.** Volcano plots illustrate significantly differentially expressed proteins (DEPs) in the (**A**). left parotid, (**B**). right parotid, and (**C**). submandibular salivary glands of xerostomic patients. The −log_10_ *p*-value (Benjamini–Hochberg corrected) is plotted against the log_2_ fold change. The vertical dotted lines denote ± 1.2 fold change, while the horizontal dotted line denotes α = 0.05. Significant hits crossing both thresholds are depicted in red, green nodes are not significant based on the *p*-value but exceed the fold change threshold, and black nodes are non-significant and do not exceed the fold change threshold. Labeled proteins have known involvement in neurodegenerative diseases.

Proteomic analysis. Across all salivary gland sites, a total of 1407 proteins were detected through proteomic analysis using nanoLC-MS/MS, and the normalized proteomic data have been provided as Supplemental Data File S1. Our statistical analysis identified 86 DEPs in the left parotid, 112 in the right parotid, and 73 in the submandibular glands of xerostomic patients. A post hoc Mann–Whitney U-test performed on protein expressions between males and females within the NX-group determined that there was no significant gender bias on DEPs after Benjamini–Hochberg FDR correction (*p* > 0.05). Volcano plots of significant proteins are presented in Figure 2. The top 20 significant proteins for each salivary gland are presented in Table 2. A complete listing is presented in Supplemental Table S2.

**Figure 3:**
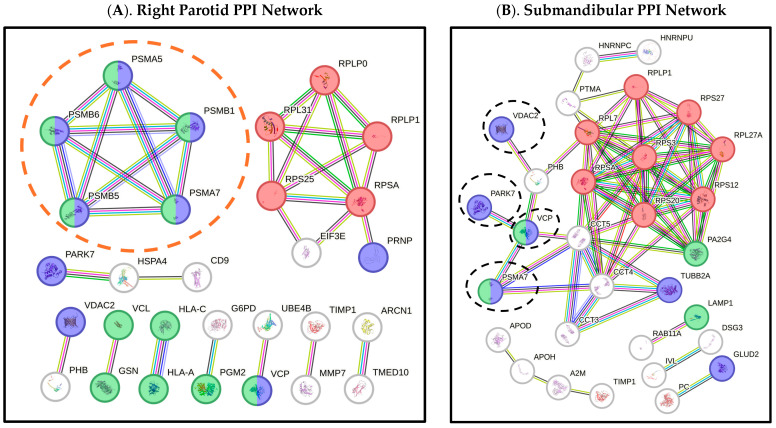
Protein–protein interaction (PPI) networks. **Legend.** The Search Tool for Recurring Instances of Neighboring Genes (STRING_v12.0_) used to create PPI networks for the (**A**). right parotid and (**B**). submandibular salivary glands. Protein–protein interaction networks created with differentially expressed proteins (DEPs) in xerostomic patients (XP-group). The (**A**). right parotid network of 29 genes (input = 112 genes) created with a confidence level of 0.90 and has an enrichment *p* = 8.86 × 10^−5^. The (**B**). submandibular network of 31 genes (input = 73 genes) created with a confidence level of 0.70 and has an enrichment *p* = 7.98 × 10^−12^. Purple nodes indicate genes associated with neurodegenerative diseases, green nodes are involved with the innate immune system, and red nodes are involved with cytoplasmic ribosomes. The orange dashed circle highlights the 20S proteasome core complex proteins, and the black dashed ellipses identify neurodegenerative disease-associated genes in the submandibular network, seen in similarity with the right parotid.

Interaction analysis. PPI networks with significant enrichment *p*-values (*p* < 0.05) at 70% and 90% confidence levels were created from DEPs of each gland site. The 112 DEPs from the right parotid gland were used as input to create a network of 29 genes with a confidence level of 0.90 and enrichment *p* = 8.86 × 10^−5^. The 73 DEPs from the submandibular gland were used to make a network of 31 genes with a confidence level of 0.70 and enrichment *p* = 7.98 × 10^−12^ (Figure 3; Supplemental Table S2). Pathway analysis identified protein networks involving several neurodegenerative diseases mainly in both the right parotid and submandibular glands (Figure 3). These networks include the downregulation of the 20S proteasome core complex and upregulation of tubulin proteins.

**Figure 4:**
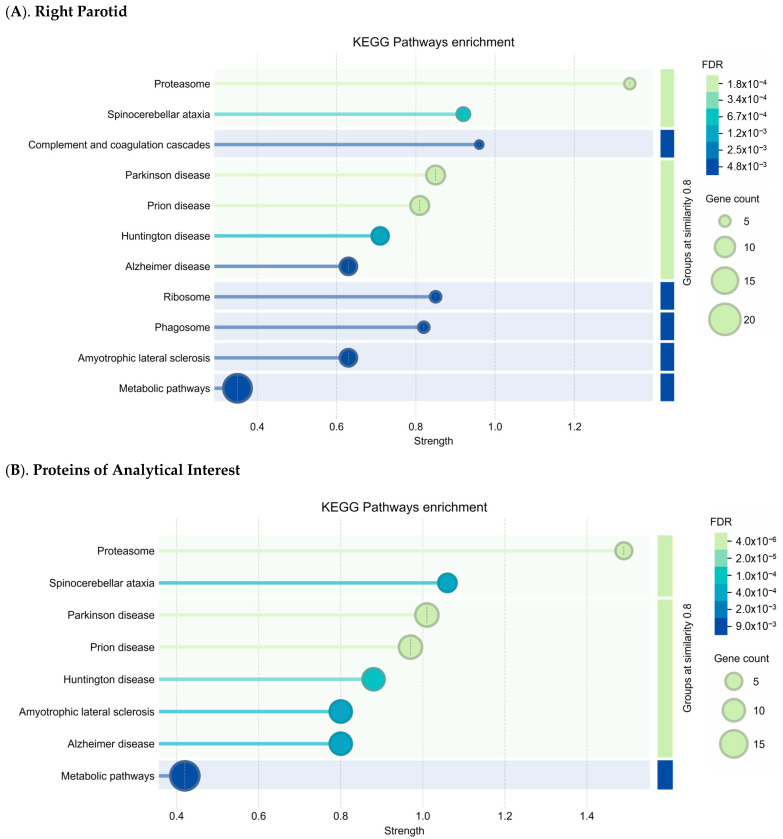
Pathway enrichment analysis results generated using the functional enrichment visualization tool in The Search Tool for Recurring Instances of Neighboring Genes (STRING_v12.0_) with the Kyoto Encyclopedia of Genes and Genomes (KEGG_v112.1_) pathways database for 112 differentially expressed proteins (DEPs) in the (**A**). right parotid and 89 DEPs from all gland sites with (**B**). proteins of analytical interest including known involvement in the oral cavity, neurological disorders, glyoxalase system, or oxidative stress. Pathway enrichment was determined by STRING_v12.0_ using a hypergeometric test for over-representation, with *p*-values corrected for multiple testing using the Benjamini–Hochberg correction for the false discovery rate (FDR). Pathways are ranked by strength, with bubble size representing the number of DEPs mapped to the pathway and bubble color representing the FDR.

The Kyoto Encyclopedia of Genes and Genomes (KEGG_v112.1_) pathways enrichment analysis of the 112 right parotid DEPs identified several protein-encoding genes, including 20S proteasome core complex proteins involved in neurological diseases such as Amyotrophic Lateral Sclerosis (ALS), Spinocerebellar Ataxia, Parkinson’s, Alzheimer’s, Huntington’s, and Prion disease (Figure 4A). Similarly, KEGG enrichment analysis of 89 DEPs of analytical interest across all three gland sites highlights the pathways linked to neurodegenerative diseases, including multiple genes involved in metabolic pathways (Supplemental Table S3; Figure 4B). Corresponding KEGG enrichment gene set tables are presented in Supplemental Table S4.

## 3. Discussion

This study provides novel insights into the salivary proteome of patients with medication-associated and autoimmune disease-associated xerostomia. The identification of DEPs in xerostomic patients highlights possible underlying mechanisms contributing to salivary gland dysfunction and potential connections to neurodegenerative conditions, including ALS, Huntington’s, Alzheimer’s, Prion, and Parkinson’s disease (Figure 2). Analysis using STRING_v12.0_ revealed these neurodegenerative pathways to be linked through notable downregulation of six 20S proteasome core complex (PSM) proteins and upregulation of two tubulinopathy-related (TUB) proteins across the major salivary glands, linking xerostomic salivary hypofunction to neuronal degeneration and impaired proteasome activity (Table 2).

In the nervous system, the proteasome is important for maintenance of cellular homeostasis in neurons, with studies highlighting its involvement with neurodevelopment and synaptic plasticity, as well as its inhibition to be a factor in neurodegenerative disease progression [24]. Proteasome impairment is a major finding in our study, evident through the downregulation of six PSM subunit proteins across all three salivary gland sites. Dysregulation of the 20S proteasome could be explained by the downregulation of Parkinson Disease (PD) protein 7 (encoded by *PARK7*; alias DJ-1) in both the right parotid and submandibular glands of xerostomic patients (Figure 3). It has been found that PD protein 7 participates in oxidative stress responses and modulates proteasome activity to control the unselective degradation of damaged proteins [25]. PD protein 7 under oxidative stress activates Nuclear respiratory factor 2 (Nrf2), an oxidative transcription regulator, which further controls the regulation of the 20S proteasome through induced expression of NAD (P) H Quinone Dehydrogenase 1 (*NQO1*). Mutations of PD protein 7 and *NQO1* are associated with familial Parkinson’s disease and Alzheimer’s disease, respectively, suggesting that symptomatic xerostomia shares mechanisms with neurodegeneration, particularly involving impaired proteasome function and regulatory responses to oxidative stress.

PD protein 7′s role in neuronal regulation and cellular defense against oxidative stress can be attributed to its function as a glyoxalase, essential for removing harmful methylglyoxal (MGO) modifications from proteins and nucleic acids [26]. Elevated MGO levels and the subsequent formation of advanced glycation end products (AGEs) may contribute to salivary gland dysfunction, while the downregulation of PD protein 7 in xerostomic patients suggests impaired MGO detoxification [27]. Importantly, PD protein 7 is secreted by salivary glands and has oral involvement at the protein level, underscoring its potential significance in maintaining salivary function. The relationship between PD protein 7, MGO, and salivary gland function also highlights the involvement of oxidative stress in xerostomia.

Complementary glyoxalase proteins identified in this study further emphasize the complexity of MGO detoxification related to xerostomia. Glyoxalase I encoded by *GLO1*, a primary glyoxalase enzyme with functions analogous to PD protein 7 and known involvement in the oral mucosa, was also downregulated in the right parotid (Table 2). Likewise, Glyoxalase I is essential to MGO detoxification and prevention of AGE formation, where its reduced expression suggests an important dysregulation of glyoxalase pathways in xerostomia patients. In contrast, Glyoxalase domain-containing 4 encoded by *GLOD4*, a paralog of Glyoxalase I, known to be expressed in salivary glands, was upregulated in the left parotid of our study and possibly represents a mechanism to compensate for MGO-induced damage (Table 2). The glyoxalase system and impaired MGO detoxification showcases the connection between type 2 diabetes and Parkinson’s disease, linking oxidative stress to the development of xerostomia [27,28]. A systematic review comparing xerostomia and salivary flow in diabetes mellitus (DM), both type 1 and type 2, versus non-DM individuals has revealed higher prevalence rates of xerostomia and lower salivary flow rates in DM patients, suggesting a role of oxidative stress and AGE formation to salivary dysfunction in DM patients [29].

The drug Gemigliptin, a dipeptidyl peptidase-4 (DPP-4) inhibitor, offers a potential therapeutic angle for MGO-induced salivary gland dysfunction. One study demonstrated that Gemigliptin inhibits AGE formation and exerts anti-glycation effects similar to the functions of PD protein 7 and Glyoxalase I in mitigating oxidative stress [27]. In rat models of MGO-induced salivary gland dysfunction, Gemigliptin was shown to significantly improve saliva flow rates, suggesting a possible treatment to restore salivary gland function in xerostomia patients [27]. Furthermore, Gemigliptin was found to aid in the decreased expression of a gene known to control salivary gland expression, i.e., Aquaporin 5 (*AQP5*), in MGO-induced rats [27].

A study by Kawedia et al. determined that Hypoxia inducible factor 1 (*HIF-1α*), the regulator of many adaptive responses to hypoxia, is a transcription factor that inhibits *AQP5* and can undergo proteasome-mediated degradation [30]. The study also showed that under normoxic conditions, the protease inhibitors stabilizing *HIF-1* downregulate *AQP5* expression in the lungs of mice [30]. Mitochondrial reactive oxygen species (ROS) increase during hypoxia and are stimulators of HIF activation, potentially leading to the downregulation of *AQP5* [31]. This mechanism aligns with our observed downregulation of the proteasome core complex in xerostomia patients, although it is unclear whether any patients included in this study were experiencing hypoxia or had a history of smoking. While *AQP5* was not detected in dried saliva samples, the observed downregulation of six 20S proteasome subunits might be explained by the fact that they can be secreted via exosomes (Table 2) [32].

Findings from a phase 1 gene therapy study utilizing an adeno-associated virus (AAV2) to deliver Aquaporin-1 (hAQP1) directly into salivary gland duct cells demonstrated the significant therapeutic potential of targeting aquaporins in xerostomic patients [33]. hAQP1 is a water channel protein that facilitates water movement across cell membranes and the delivery of hAQP1 via AAV2 in this study resulted in notable improvements in salivary flow rates and reduced xerostomia symptoms in patients with salivary gland dysfunction [33]. These findings underscore aquaporins, such as AQP1 and AQP5, as promising targets for the effective treatment of xerostomia.

Downregulation of Glutamate dehydrogenase 2 encoded by *GLUD2* in submandibular glands observed in our study suggests additional metabolic dysregulation in xerostomia patients (Table 2). Glutamate dehydrogenase 2 recycles glutamate during neurotransmission and catalyzes its conversion to alpha-ketoglutarate, a key metabolite in the production of cellular energy. Voltage-dependent anion channel 2, encoded by *VDAC2*, located in the outer mitochondrial membrane, involved in regulating the apoptosis of dopaminergic neurons, was also downregulated in the submandibular gland but upregulated in the right parotid (Table 2; Figure 3). Notably, both *GLUD2* and *VDAC2* encoded proteins have oral involvement in salivary glands and are associated with Parkinson’s disease, emphasizing metabolic complexity and its connection to neurodegenerative disorders in salivary gland dysfunction [34,35]. However, neither protein is known to be directly secreted in saliva, suggesting that the upregulation of VDAC2 in the right parotid may be explained by mitochondrial or salivary gland damage, as non-secreted proteins potentially leak into the duct orifice, where we used paper strips to collect saliva samples. Alternatively, as mentioned earlier, proteins can be secreted via exosomes and not directly secreted in saliva (Table 2).

Ubiquinol-cytochrome c reductase core protein 1 (encoded by *UQCRC1*) is an additional protein downregulated in the right parotid, highlighting mitochondrial dysfunction to neurodegenerative diseases in relation to xerostomia. UQCRC1 encodes a subunit of mitochondrial respiratory chain complex III, important for proper mitochondrial function, ATP synthesis, and neuronal morphogenesis [36]. Our pathway analysis identified this protein to be associated with neurodegenerative diseases in similarity to the proteasome and tubulin proteins (Supplemental Table S4). Akin to VDAC2, UQCRC1 emphasizes mitochondrial dysregulation to the progression of neurodegenerative diseases through dopaminergic neuronal degeneration, possibly related to the development of xerostomia.

The downregulation of Unc-5 Netrin Receptor C (encoded by *UNC5C*) in the right parotid (Table 2) further implies neuronal regulation in xerostomia. Unc-5 Netrin Receptor C, a transmembrane receptor involved in neuronal apoptosis regulation and axonal guidance, was not detected in our PPI networks or pathways enrichment but is found to be implicated in both Alzheimer’s and Parkinson’s diseases [37,38]. However, UNC5C secretion by the salivary glands or oral involvement at the protein level remains unknown. In Parkinson’s disease, UNC5C is cleaved by the protease Asparagine Endopeptidase (AEP), contributing to dopaminergic neuronal loss. AEP-truncated UNC5C fragmentation promotes neurotoxicity and the aggregation of alpha-synuclein, observed in brains of mouse models for Parkinson’s disease [39]. Besides PD protein 7, downregulation of Unc-5 Netrin Receptor C in xerostomia patients is also in favor of a possible neuronal dysregulation associated with salivary gland hypofunction.

Further neuronal implications to xerostomia can be connected by dysfunctions of the tubulin cytoskeleton, known to share associations with several of the neurodegenerative diseases found in our pathway analysis involving proteasome dysregulation (Supplemental Table S4B) [40]. Microtubules are the main components of the cytoskeleton, important for cell shape modulation and intracellular organization of the cytoplasm, consisting of both α and β tubulin subunits [40]. Tubulin alpha 4a, encoded by *TUBA4A,* and Tubulin beta 2a, encoded by *TUBB2A,* were upregulated in the right parotid and submandibular glands, respectively, and are involved in microtubule-mediated axon outgrowth and maintenance [41]. Mutations of these genes being identified in neurodegenerative diseases can be attributed to defects in axonal transport, implicating shared molecular features between xerostomia and microtubule deficiency.

The present study provides an understanding of the molecular mechanisms underlying salivary gland dysfunction in patients with medication-associated and autoimmune disease-associated xerostomia. The downregulation of key proteins involved in oxidative stress and neurodegenerative diseases, encoded by *PARK7*, *GLO1*, *GLUD2*, *VDAC2, UQCRC1,* and *UNC5C,* including the 20S proteasome core complex proteins, highlights a possible mechanistic link to xerostomia pathophysiology. KEGG pathway enrichment further expands these conclusions by illustrating the neurodegenerative diseases associated with the DEPs identified in this study (Figure 4). The right parotid exhibits many DEPs related to neurological conditions such as ALS, Alzheimer’s, Parkinson’s, Huntington’s, and Prion disease (Figure 4A). Figure 4B further highlights neuronal and metabolic-related DEPs across all salivary glands, suggesting a link between mitochondrial dysregulation, neurodegenerative diseases, and salivary gland dysfunction in xerostomia patients.

Through proteomic profiling of xerostomic patients, we determined how the dysregulation of key biological pathways, including glyoxalase detoxification and aquaporin-mediated fluid transport, is likely to contribute to salivary gland dysfunction. Current therapies on anti-glycation and aquaporin expression reinforce these associations to xerostomia based on our proteomic analysis. Additionally, neurodegenerative disease testing, such as needle biopsies of salivary glands, remains invasive, costly, and complicated. In contrast, salivary diagnostics can potentially serve as an early non-invasive detection tool for neurological dysfunction. Due to the high variability of protein expressions across populations, the increased standardization of saliva collection and testing methods and a larger cohort with appropriate patient stratification are necessary to confirm our findings prior to clinical translation. Overall, our study demonstrates that salivary biomarkers show promise for accessible screening of diseases, with the potential to be a precise and scalable diagnostic tool for personalized therapeutic interventions.

## 4. Methods

Patient cohort. A total of 45 patients (18 non-xerostomic and 27 xerostomic) were recruited and first stratified based on being healthy control subjects (*n* = 6), control subjects with comorbidity (*n* = 12), case polypharmacy patients with comorbidity (*n* = 8), case patients with comorbidity and primary autoimmunity (*n* = 15), and case patients with comorbidity and other autoimmunity (*n* = 4). Control subjects with or without comorbidity were grouped together as the non-xerostomic subjects (NX-group; *n* = 18), and xerostomic case patients with comorbidity and any autoimmunity were grouped as xerostomic patients (XP-group; *n* = 27). Non-xerostomic subjects consisted of 5 males and 13 females, whereas the xerostomic patient group consisted of females only. The study was approved by the institutional review board at Atrium Health-Wake Forest Baptist (IRB Study #IRB00083131). All patients signed an informed consent form.

Sample collection. Subjects were instructed to rinse their mouth for 1 min with water and spit. Salivary gland orifices (left and right parotid and submandibular glands) were isolated using dental cotton rolls (Stensen’s duct for parotid collection and Wharton’s duct for submandibular gland collection) and gland areas were dried, skipping the first saliva secretion. Dried saliva spot samples were collected using PerioCol Paper Saliva Collection Strips (Oraflow Inc., Smithtown, NY, USA) for the three isolated salivary glands for all 45 patients, totaling 135 samples suitable for proteomics analysis [42]. The strips were applied mid-stream to prevent contact with oral mucosa, and secretions were collected for 30 s. Samples were dried for 1 h at room temperature and stored at −80 °C until further analysis. Additionally, unstimulated and stimulated salivary flow rates were measured for correlation testing and the identification of outliers in each patient group.

Proteomic Analysis. Dried saliva spot samples (~2 µL volume collected) [42] were prepared using in-StageTip (iST) Sample Preparation kits. In total, 50 µL of lyse buffer was added to the samples and heated at 95 °C for 10 min at 1000 rpm with agitation. After cooling to room temperature, trypsin digestion buffer was added to the samples and incubated at 37 °C for 2 h at 500 rpm with shaking, and halted by the addition of a stopping buffer. Sample clean-up and desalting were carried out in the iST cartridge with wash buffers, followed by peptide elution with 2 × 100 µL elution buffer, and then lyophilized by SpeedVac.

A spectrum library was established using an aliquot of pooled samples. The peptide mixture was re-dissolved in buffer A containing H_2_O at pH 10.0 adjusted with ammonium hydroxide, then fractionated by high pH separation using the nanoACQUITY UPLC system (Waters Corporation, Milford, MA, USA) connected to a reverse phase column (C18 column, 2.5 mm × 250 mm, 1.9 µm). High pH separation was performed using a non-linear gradient with buffer B containing 80% acetonitrile at pH 10.0 adjusted with ammonium hydroxide, starting at 4% to 53% in 40 min, 53% to 68% in 15 min, and 68% to 95% in 5 min, and then maintained for 3 min. The column was re-equilibrated at the initial condition for 17 min, while the flow rate was maintained at 2 µL/min and the temperature was maintained at 30 °C. Ten factions were collected with the EasyPept Frac nano automatic fraction collection system, each of which was dried in a vacuum concentrator for data collection.

The data were collected using the UltiMate 3000 (Thermo Fisher Scientific, Waltham, MA, USA) liquid chromatography system connected to the timsTOF Pro 2 (Bruker Daltonics, Billerica, MA, USA) ion-mobility spectrometer for tandem mass spectrometry (LC-MS/MS) using both Data-Dependent Acquisition (DDA) and Data-Independent Acquisition (DIA) methods. Samples were reconstituted in 0.1% formic acid and 200 ng of peptides were separated by IonOpticks analytical columns of 15 cm length, 75 µL i.d, 1.7 µm particle size, and 120 Å pore size. Gradient staining was then performed for 60 min with a buffer containing 80% acetonitrile with 0.1% formic acid, starting at 4% followed by a stepwise increase to 28% in 25 min, 44% in 10 min, and 90% in 10 min and maintained for 7 min, and finally equilibrated at 4% for 8 min. The column flow rate was maintained at 400 nL/min with a temperature of 50 °C.

For DDA mass spectrometry, the instrument operated in DDA PASEF mode with 10 PASEF scans per topN acquisition cycle using an accumulation and ramp time of 100 ms each. MS and MS/MS spectra were recorded from 100 to 1700 *m*/*z* with an ion mobility range (1/K0) of 0.6–1.6 Vs/cm^2^. The included charge was set to 0–5, the target value was set to 10,000, and dynamic exclusion was set to 0.4 min. The quadrupole isolation width was set to 2 Th for *m*/*z* < 700 and 3 Th for *m*/*z* > 700. For DIA mass spectrometry, the instrument was operated in diaPASEF mode with 22 × 40 Th precursor isolation windows from *m*/*z* 349 to 1229, using a 13-scan diaPASEF scheme with repetition of steps 2–5 to adapt the MS1 cycle time. During scanning, the collision energy was ramped linearly as a function of the mobility from 59 eV at 1/K0 = 1.6 Vs/cm^2^ to 20 eV at 1/K0 = 0.6 Vs/cm^2^.

Raw DDA and DIA data were processed with the Spectronaut 18 (Biognosys AG, Schlieren, Switzerland) system under default settings with the ‘Homo_sapiens’ Uniprot database. Trypsin was set as the digestion enzyme with carbamidomethyl on cysteine as a fixed modification and oxidation on methionine specified as the variable modifications, with the DDA method also having acetylation on Protein N-terminus as the variable modifications. A 1% Q-value false discovery rate cutoff was applied on both the precursor and protein levels. In DIA analysis, the retention time prediction type was set to dynamic iRT, and data extraction was determined dynamically by Spectronaut based on extensive mass calibration. Decoy generation was set to mutate, which will apply a random number of AA position swaps of min = 2 and max = length/2. Normalization was performed using a local normalization strategy, and peptides that passed the 1% Q-value cutoff were used to calculate the major group quantities MaxLFQ method. Due to the substantial variability across dried saliva spot samples, data preprocessing was performed to retain data from groups with quantifiable measurements and biologically relevant signals. Data preprocessing included contaminant removal and handling of missing values, where proteins missing in over 50% of samples within a group were considered non-expressed, while those missing in 50% or less of samples within a group were imputed with the minimum value (Creative Proteomics, Shirley, NY, USA).

Briefly, DDA of the pooled samples involves performing a full MS scan to detect peptide precursor ions, followed by selecting the most abundant ions (typically the top 10–20) for fragmentation in MS/MS scans to establish a spectrum library. In contrast, DIA applied to each individual sample systematically fragments all ions within a predefined *m*/*z* window across the entire mass range, generating a more complex spectrum due to the simultaneous fragmentation of multiple precursor ions. Since the dried saliva spot samples contained low protein amounts, both the DDA and DIA methods were used to maximize the coverage and quality of proteomic data.

Statistical and interaction analysis. Following total intensity normalization, differentially expressed proteins (DEPs) were identified in the XP-group relative to the NX-group using the Mann–Whitney U-test, with Benjamini–Hochberg FDR correction (*p*-adjusted (*p*_adj_) < 0.05). DEPs with fold changes >1.2 were considered upregulated, while <1.2 were considered downregulated. Fold changes were calculated as the relative expression difference, using the formula (test-control)/control. For downregulated proteins, fold change values were expressed as the reciprocal to facilitate comparison with upregulated proteins. This is a pilot study. Using the wmwpow_v0.1.3_ package in R for empirical power analysis of the Mann–Whitney U tests [43], we determined that about 20% of detected proteins across the salivary glands achieved an acceptable power exceeding 70%. None of the proteins below that level were discussed. Volcano plots were plotted for each gland site using RStudio_v2023.06.1_ with the EnhancedVolcano_v1.18.0_ package [44]. The STRING_v12.0_ database was used to construct protein–protein interaction (PPI) networks from DEPs at each gland site [45]. PPI enrichment *p*-values (unadjusted) were calculated by STRING_v12.0_ using a hypergeometric test to assess the likelihood that observed interactions among input proteins are significantly enriched by random chance [46]. Pathway enrichment analysis was conducted using the KEGG_v112.1_ database within STRING_v12.0_, and results were plotted using the functional enrichment visualization tool [47]. The enrichment of KEGG pathways was assessed using a hypergeometric test, evaluating if the observed number of DEPs in each pathway is significant by chance and based on the total number of proteins in the pathway. *p*-values were adjusted for multiple testing using the Benjamini–Hochberg procedure for the false discovery rate (FDR).

## 5. Limitations

While our findings provide important insights into xerostomia pathophysiology, several limitations should be considered, including sample size and confirmation with an independent repeat cohort. Due to the unintentional absence of males in the xerostomia patient group that occurred during the actual recruitment period for our study cohort, it must be noted that these results cannot reliably be generalized across genders. In the future, we aim to recruit a more diverse sample, including males, to confirm our findings. Furthermore, subjective perception of dry mouth can vary between patients, making xerostomia more difficult to quantify and categorize. Additionally, differences in comorbidities and the inability to control for polypharmacy may have impacted the results.

## 6. Conclusions

This study identified potential critical protein dysregulation in symptomatic xerostomia associated with medications and autoimmune diseases, linking to pathways involved in mitochondrial dysfunction, oxidative stress, and several neurological disorders. Reduced expression of PD protein 7 in major salivary glands of xerostomic patients suggests impaired methylglyoxal (MGO) detoxification and a possible deficiency in parasympathetic nervous system innervation, contributing to salivary gland dysfunction. Aquaporin-targeted therapies, exemplified by the promising effects of Gemigliptin in anti-glycation and AQP5 upregulation, also hold potential to restore salivary gland function. Further studies are needed to validate these pathways and develop therapeutic interventions to alleviate symptoms in xerostomic patients.

## Figures and Tables

**Table 1:** Patient demographics and group distribution.

Group ^a^	# Patients ^b^	M/F ^c^	Age (Min./Max./SD) ^d^	Age (avg) ^e^	Ethnicity (AA/Caucasian/Other) ^f^
NX-group ^g^	18	5/13	31/82/12.74	57.89	3/10/5
XP-group ^h^	27	0/27	48/79/6.82	64.41	1/26/0

**^a^** Patient groups. **^b^** Number of patients in each group. **^c^** Ratio of male to female patients. **^d^** Minimum (Min.), maximum (Max.), and standard deviation (SD) of age (years). **^e^** Average age (years). **^f^** Patient ethnicity categorized as African American (AA), Caucasian, or Other. **^g^** Non-xerostomic control subjects (NX-group). **^h^** Xerostomic patients (XP-group).

**Table 2:** Top 20 differentially expressed proteins (DEPs).

Gene Name ^a^	Protein Name ^b^	Direct Saliva Secretion ^c^	Exosome (Indirect Secretion) ^d^	Oral Involvement ^e^	Salivary Gland/s ^f^	Up/Down-Regulated ^g^	Fold Change ^h^
*DSG3*	Desmoglein 3	No	Yes	Yes	LP; RP; SMSL	Up; Down; Down	35.90; 32.33; 37.23
*GLO1*	Glyoxalase I	No	Yes	Yes	RP	Down	14.06
*GLOD4*	Glyoxalase Domain Containing 4	No	Yes	Yes	LP	Up	26.63
GLUD2 *	Glutamate Dehydrogenase 2	Unknown	No	Yes	SMSL	Down	43.00
*MUC1*	Mucin 1	No	Yes	Yes	LP	Down	18.10
*MUC7*	Mucin 7	Yes	No	Yes	LP	Up	132.36
PARK7 *	Parkinson Disease Protein 7	Yes	No	Yes	RP; SMSL	Down; Down	25.58; 47.43
PRNP *	Prion Protein	No	Yes	Yes	RP	Down	8.92
PSMA5 *	Proteasome 20S Subunit Alpha 5	No	Yes	Yes	RP	Down	28.26
PSMA6 *	Proteasome 20S Subunit Alpha 6	No	Yes	Yes	LP	Down	14.64
PSMA7 *	Proteasome 20S Subunit Alpha 7	No	Yes	Yes	RP; SMSL	Down; Down	11.79; 11.36
PSMB1 *	Proteasome 20S Subunit Beta 1	No	Yes	Yes	RP	Down	12.80
PSMB5 *	Proteasome 20S Subunit Beta 5	No	Yes	Yes	RP	Down	5.46
PSMB6 *	Proteasome 20S Subunit Beta 6	No	Yes	Yes	RP	Down	20.96
TUBA4A *	Tubulin Alpha 4a	No	Yes	Yes	RP	Up	23.67
TUBB2A *	Tubulin Beta 2a	No	Yes	Yes	SMSL	Up	1.16
UNC5C *	Unc-5 Netrin Receptor C	Unknown	Unknown	Unknown	RP	Down	28.03
UQCRC1 *	Ubiquinol-Cytochrome C Reductase Core Protein 1	Unknown	Unknown	Yes	RP	Down	11.27
VCP *	Valosin Containing Protein	No	Yes	Yes	LP; RP; SMSL	Up; Up; Down	10.49; 10.75; 6.79
VDAC2 *	Voltage Dependent Anion Channel 2	No	Yes	Yes	RP; SMSL	Up; Down	8.04; 13.63

**Footnote.** The top 20 DEPs in xerostomic patients (XP-group) were selected based on known involvement with either the oral cavity, neurological disorders, or glyoxalase system. **^a^** Entrez gene name. **^b^** Protein name. **^c^** Known to be directly secreted in saliva by salivary glands. **^d^** Presumed to be indirectly secreted in saliva through exosomes. **^e^** Known oral involvement at the protein level in either the oral mucosa or salivary glands. **^f^** Salivary glands: left parotid (LP), right parotid (RP), and submandibular (SMSL). **^g^** Upregulation or downregulation of genes in the salivary gland. **^h^** Relative fold change. Note: genes implicated in neurodegenerative diseases are depicted using an asterisk (*).

## Data Availability

All supporting files can be found on our lab’s Github page (www.github.com/mbeckm01/Xero_Proteomics accessed on 18 July 2025).

## Supplementary Materials

The following supporting information can be downloaded at: https://www.mdpi.com/article/10.3390/ijms26157037/s1.
