# Supplementary material for: Salivary Proteome Profile of Xerostomic Patients Reveals Pathway Dysregulation Related to Neurodegenerative Diseases: A Pilot Study
Source: Int J Mol Sci. 2025 Jul 22;26(15):7037. doi: 10.3390/ijms26157037 (PMC12346731; doi:10.3390/ijms26157037)
Supplement: Supplementary file 1 [file ijms-26-07037-s001.zip › Xero Proteomics- Supplemental Table S1. Comorbidities and Medications.pdf]

Supplemental Table S1: Comorbidities and Medications

Supplemental Table S1A. List of Comorbidities

**NX-group**[a]	**XP-group**[b]
Anxiety disorder	Allergic rhinitis
Arthritis	Anemia
Asthma	Anxiety disorder
Cardiovascular disease	Arthritis
GERD	Asthma
Gilbert syndrome	Atherosclerosis
Hypertension	Autoimmune disease
Melanoma	Back pain
Migraines	Barretts esophagus
Temporomandibular disorders (TMDs)	Bronchitis
Diabetes Mellitus	Cardiomyopathy
Hypertension	Chronic obstructive pulmonary disease (COPD)
Hyperthyroidism	Chronic pruritus
Hypolipidemia	Clotting disorder
Osteoarthritis	Common variable immunodeficiency
Pre-diabetis	Connective tissue disease
Pre-hypertension	Depression
Pre-menopause	Diabetes mellitus
Renal disease	Disc degeneration
	Diverticular disease
	Dry eye syndrome
	Dyslipidemia
	Epstein Barr Virus infection
	Fibromyalgia
	GERD
	GVHD
	Hearing loss
	Heart murmurs
	Hemolytic anemia
	Hepatitis-autoimmune
	Herpes
	Hiatal hernia
	History of TB
	HLAB27
	Hypercholesterolemia
	Hypertension
	Hyperthyroidism
	Hypoparathyroidism
	Hypothyroidism
	Inflammatory polyarthritis
	Interstitial cystitis
	Iron deficiency

	Irritable bowel syndrome (IBS)<br>Lichen planus<br>Lower back pain<br>Lung disease<br>Lupus*<br>Menopause<br>Metabolic syndrome<br>Mild hyperlipidemia<br>Mitral valve prolapse<br>Multinodular goiter<br>Myalgia<br>Nephrolithiasis<br>Non-allergic rhinitis<br>Non-HIV STD<br>Non-HNC cancer<br>Non-oral candidiasis<br>Obesity<br>Ocular migraine<br>Oral lichen planus (OLP)<br>Osteoarthritis<br>Osteopenia<br>Osteoporosis<br>Pectus excavatum<br>Pericarditis<br>Peripheral neuropathy<br>Polymyositis<br>Raynaud's<br>Reflux<br>Restless leg syndrome<br>Rheumatic heart disease<br>Rheumatoid arthritis*<br>Rosacea<br>Sciatica<br>Scleroderma<br>Sicca*<br>Sinusitis<br>**Sjogren's Syndrome**<br>Sleep apnea<br>Spondylarthritis<br>Stomatitis<br>Temporomandibular disorders (TMDs)<br>Thoracic aortic aneurysm<br>Thrombosis of the brachiocephalic vein<br>Thyroid nodule<br>Thyroiditis<br>Urinary incontinence<br>Urinary tract infection<br>Uveitis

	Vertigo<br>VitB12 deficiency<br>VitD deficiency

Supplemental Table S1B. List of Medications

NX-group[a]	XP-group[b]
Advair-diskus	Acidophilus probiotic blend
Alaritin	Adderall
Alprazolam (PRN)	Advair
Amlodipine	Albuterol
Amlopodene	Amaryl
Asmanex	Ambien
Atorvastatin	Antivent
Atrovent nasal	Atorvastatin
Bystolic	Azelastine
Claritin	Buspar
Cyclobenzaprine	Bystolic
Ezetimibe	Ca+ carbonate
Flonase	Capsaicin
Gabapentin	Carbamazepine
Glipizide	Celebrex
Irbesartan	Celecoxib
Levothyroxine	Celexa
Lexapro	Cevimeline
Lisinopril HCTZ	Citrucel
Losartan HCTZ	Claritin
Magic Mouthwash	Clebrex
Metformin	Clobetasol
Metoprolal	Clobetasol topical
Nexplanon implant in arm	Clotrimalzole topical
Omeprazole	Colace
Pepaid	Colestid
Pravastatin	Cozaar
Ramipril	Crestor
Rosuvastatin	Cymbalta
Serevent Diskus	Deltasone
Simvastatin	Dexilant
Singular	Dichofenac sodium
Sitagliptin	Diflucan

Stor<br>Temovate cream<br>Timolol drops<br>Tizanidine<br>Tylenol<br>Tyleyesl<br>Valium<br>Ventolin<br>Xanax<br>Zocor<br>Zyrtec	Diphenhydramine HCL inj<br>Dronabinol<br>Dulera<br>Duloxetine<br>DuoNeb Benlysta<br>Effexor<br>EpiPen<br>Estradiol patch<br>Estradiol topical<br>Evozac<br>Extradiol patch<br>Famotidine<br>Fexofenadine<br>Flexeril<br>Flonase<br>Fluocinonide<br>Fluodinonide<br>Fluphenazine Hydrochloride (FL2)<br>Fluticasone<br>Flutidasone<br>Fosamax<br>Gabapentin<br>Gammaplex<br>HCTZ<br>Hycodan<br>Hydodan<br>Hydrochlorothiazide<br>Hydroquinone topical<br>Hydroxychloroquine<br>Hydroxycholorquine<br>Hydroxyzine<br>Hyoscyamine<br>Imitrex<br>Imodium<br>Insulin<br>Iovastan<br>Iron<br>Iron prilosec<br>Keppra<br>Ketoconazole cream<br>Ketodonazole dream<br>Ketorolac<br>Kiazepam<br>Klonopin<br>Lactobac<br>Lamictal<br>Levalbuterol<br>Levothyroxine<br>Lexapro<br>Lifitegrast ophthalimic

Lipitor
Lisinopril
Lorazepam
Losartan
Lovastatin
Lycopene
Magic Mouthwash
MBX rinse
Meclizine
Meloxicam
Metformin
Micarditis
Midamor
Mirena
Mirtazapine
Movantik
Mycelex troche
Nabumetone
Naproxen rizatriptan
Nasal spray
Neoprene patch
Neurontin
Newrontin
Nexium
Novofine
Nulev
Nystatin
Omnicef
Oxybutynin
Ozempic
Pantoprazole
Pepcid
Percocet
Phenergan
Phentermine
Pilocarpine
Pioglitazone
Plaquenil
Prair HFA90
Pranimpexole
Pravachol
Prednisone
Premarin
Prevident 5000
Prilosec
Privigen
Pro Air HFA
Pro air HFA90
Probiotic
Progesterone
Protonix

Proventil
Pulmicort
Refresh digital opht
Remicaide
Restasis
Restoril
Rosuvastatin
Senna-gen
Sertraline
Setrakubem gabapentin
Simvastatin
Singular
Symbicort
Synthroid
Tenormin
Testosterone
Timolol
Tinactin
Tinadtin
Tizanidine
Tobradex
Topamax
Trazodone
Triamcinolone
Triamterene
Trizanicine
Tylenol
Valacyclovir
Valium
Vaniqua topical
Venlafaxine
Vistari
Vit B12
Vit D
Vit D3
Vitamin B12
Vitamin D3 2000
Voltaren
Voltavren
Warfin
Wellbutrin
Xanax
Xlidra
Xopenex
Xylocaine
Zanaflex
Zantac
Zantad
Zetta
Zocor
Zofran

	Zolipiden<br>Zomig-amt<br>Zyrtec

Footnote. Complete list of **1A.** comorbidities and **1B**. medications reported by participants across each group.

[a]Non-xerostomic control subjects (NX-group)
[b]Xerostomic patients (XP-group)

Note: Within the XP-group in **1A**, primary autoimmunity associated with xerostomia is highlighted in **bold** and other autoimmune conditions causing xerostomia are depicted using asterisk (*)
