# Supplementary material for: Salivary Proteome Profile of Xerostomic Patients Reveals Pathway Dysregulation Related to Neurodegenerative Diseases: A Pilot Study
Source: Int J Mol Sci. 2025 Jul 22;26(15):7037. doi: 10.3390/ijms26157037 (PMC12346731; doi:10.3390/ijms26157037)
Supplement: Supplementary file 1 [file ijms-26-07037-s001.zip › Xero Proteomics- Supplemental Table S2. DEPs.pdf]

Supplemental Table S2: All Differentially Expressed Proteins (DEPs)

Supplemental Table S2A. Left Parotid DEPs

**Gene Name**[a]	**Up/Down-Regulated**[b]	**Fold Change**[c]	**p-value**[d]
IGKV1D-13	Up	185.66	$2.89x10^{-8}$
MUC7	Up	132.36	$3.18x10^{-8}$
SERPINB1	Up	81.74	$2.89x10^{-8}$
SFN	Up	63.37	$2.89x10^{-8}$
PRSS27	Up	55.14	$2.89x10^{-8}$
PLS3	Up	45.31	$2.94x10^{-8}$
COL6A1	Up	44.74	$2.89x10^{-8}$
LGALS7B	Up	39.30	$2.95x10^{-8}$
ARF3	Up	37.69	$2.89x10^{-8}$
SPINK5	Up	36.08	$2.89x10^{-8}$
DSG3	Up	35.90	$2.89x10^{-8}$
ANXA3	Up	33.05	$2.89x10^{-8}$
SYNCRIP	Up	31.70	$3.18x10^{-8}$
A2M	Up	29.03	$2.94x10^{-8}$
HEBP2	Up	28.73	$2.89x10^{-8}$
UBE2V1	Up	28.67	$2.89x10^{-8}$
GOT1	Up	27.33	$3.15x10^{-8}$
GLOD4	Up	26.63	$3.08x10^{-8}$
CALML5	Up	25.02	$2.89x10^{-8}$
NPEPPS	Up	24.78	$2.89x10^{-8}$
RAB7A	Up	22.11	$2.95x10^{-8}$
CDH1	Up	20.72	$2.89x10^{-8}$
SH3BGRL	Up	20.46	$2.95x10^{-8}$
RPL7	Up	20.39	$2.89x10^{-8}$
CBR1	Up	20.20	$2.89x10^{-8}$
GNG3	Up	19.78	$2.89x10^{-8}$
SBSN	Up	19.54	$2.95x10^{-8}$
CAPN1	Up	18.80	$2.94x10^{-8}$
TMED10	Up	18.15	$2.89x10^{-8}$
TYMP	Up	17.45	$2.89x10^{-8}$
IVL	Up	16.79	$2.89x10^{-8}$
GGCT	Up	16.48	$2.89x10^{-8}$
HPRT1	Up	16.27	$2.89x10^{-8}$
GAA	Up	15.41	$2.89x10^{-8}$
EEF1G	Up	14.41	$2.89x10^{-8}$
CANX	Up	13.68	$3.15x10^{-8}$
ANP32E	Up	13.67	$2.89x10^{-8}$
BANF1	Up	13.26	$2.89x10^{-8}$
SERBP1	Up	12.74	$2.89x10^{-8}$
PRKCSH	Up	12.42	$2.94x10^{-8}$
CCT6A	Up	11.63	$2.89x10^{-8}$
PNP	Up	11.36	$2.89x10^{-8}$
VCP	Up	10.49	$2.89x10^{-8}$
PDCD6IP	Up	10.45	$2.89x10^{-8}$
PPA1	Up	10.19	$2.95x10^{-8}$
CCT5	Up	9.01	$2.94x10^{-8}$
CCT7	Up	8.30	$2.89x10^{-8}$
ARPC2	Down	8.12	$2.48x10^{-9}$
RPS2	Down	8.18	$2.48x10^{-9}$
ECH1	Down	10.23	$2.48x10^{-9}$
LGMN	Down	11.71	$2.48x10^{-9}$
MARCHF8	Down	13.14	$2.48x10^{-9}$
HNRNPH3	Down	14.12	$2.48x10^{-9}$
PSMA6	Down	14.64	$2.48x10^{-9}$
IL6ST	Down	14.94	$2.48x10^{-9}$
CPD	Down	15.05	$2.48x10^{-9}$

CTSH	Down	15.18	$2.48x10^{-9}$
SLC1A5	Down	15.87	$2.48x10^{-9}$
IGF2	Down	17.12	$2.48x10^{-9}$
MUC1	Down	18.10	$2.48x10^{-9}$
PLEC	Down	19.68	$2.48x10^{-9}$
SPATA20	Down	20.50	$2.48x10^{-9}$
ENPP4	Down	21.58	$2.48x10^{-9}$
HNRNPD	Down	21.70	$2.48x10^{-9}$
QSOX1	Down	21.96	$2.48x10^{-9}$
SLC38A10	Down	24.19	$2.48x10^{-9}$
PAM	Down	26.29	$2.48x10^{-9}$
CTNNA3	Down	30.33	$2.48x10^{-9}$
ERP29	Down	31.84	$2.48x10^{-9}$
MELTF	Down	33.58	$2.48x10^{-9}$
IGLV2-18	Down	33.62	$2.48x10^{-9}$
CARD9	Down	33.84	$2.48x10^{-9}$
EGF	Down	36.20	$2.48x10^{-9}$
PROM1	Down	36.31	$2.48x10^{-9}$
IGLV5-45	Down	37.59	$2.48x10^{-9}$
CREG1	Down	40.46	$2.48x10^{-9}$
CFH	Down	44.44	$2.48x10^{-9}$
FXYD2	Down	60.16	$2.48x10^{-9}$
MMP7	Down	62.74	$2.48x10^{-9}$
FURIN	Down	64.66	$2.48x10^{-9}$
CUTA	Down	65.23	$2.48x10^{-9}$
FOLR1	Down	66.07	$2.48x10^{-9}$
GSTA1	Down	76.92	$2.48x10^{-9}$
ZNF208	Down	95.91	$2.48x10^{-9}$
SIL1	Down	113.03	$2.48x10^{-9}$
IGHV5-10-1	Down	146.92	$2.48x10^{-9}$

Supplemental Table S2B. Right Parotid DEPs

Gene Name[a]	Up/Down-Regulated[b]	Fold Change[c]	p-value[d]
TIMP1	Up	467.27	$2.47x10^{-8}$
UGT3A1	Up	118.72	$2.61x10^{-8}$
C1R	Up	114.75	$2.47x10^{-8}$
IGLV7-46	Up	84.43	$2.47x10^{-8}$
ZNF208	Up	56.35	$2.51x10^{-8}$
GNG3	Up	50.71	$2.47x10^{-8}$
ABHD14B	Up	42.80	$2.47x10^{-8}$
IGLV5-45	Up	39.23	$2.47x10^{-8}$
IGLV3-1	Up	36.58	$2.47x10^{-8}$
PAM	Up	33.26	$2.51x10^{-8}$
SPARCL1	Up	31.81	$2.51x10^{-8}$
ANPEP	Up	28.98	$2.47x10^{-8}$
RAB10	Up	28.53	$2.47x10^{-8}$
IDUA	Up	25.31	$2.47x10^{-8}$
WDR1	Up	25.07	$2.71x10^{-8}$
CAP1	Up	24.65	$2.71x10^{-8}$
CFB	Up	24.55	$2.47x10^{-8}$
TUBA4A	Up	23.67	$2.47x10^{-8}$
GSN	Up	20.14	$2.47x10^{-8}$
RPLP1	Up	20.03	$2.51x10^{-8}$
RPL31	Up	17.49	$2.47x10^{-8}$
HEXA	Up	16.30	$2.47x10^{-8}$
HLA-A	Up	15.70	$2.47x10^{-8}$
IMUP	Up	15.17	$2.47x10^{-8}$
LMAN2	Up	15.06	$2.47x10^{-8}$
NEU1	Up	13.93	$2.47x10^{-8}$
RCN1	Up	13.56	$2.61x10^{-8}$
RAP1B	Up	13.36	$2.51x10^{-8}$
BPIFA1	Up	12.78	$2.47x10^{-8}$
RPLP0	Up	12.11	$2.47x10^{-8}$
COL6A2	Up	11.92	$2.47x10^{-8}$
VCP	Up	10.75	$2.47x10^{-8}$
ACTR3	Up	9.14	$2.47x10^{-8}$
UBE2N	Up	8.98	$2.61x10^{-8}$
CCT3	Up	8.83	$2.51x10^{-8}$
FASN	Up	8.19	$2.47x10^{-8}$
VDAC2	Up	8.04	$2.47x10^{-8}$
UBE4B	Up	7.64	$2.51x10^{-8}$
CEACAM5	Up	7.05	$2.47x10^{-8}$
LGALS1	Up	6.23	$2.47x10^{-8}$
PSMB5	Down	5.46	$1.66x10^{-9}$
LYPD3	Down	6.82	$1.66x10^{-9}$
PRNP	Down	8.92	$1.66x10^{-9}$
VCL	Down	9.09	$1.66x10^{-9}$
PTBP1	Down	10.04	$1.66x10^{-9}$
PGM2	Down	10.89	$1.66x10^{-9}$
RPSA	Down	10.96	$1.66x10^{-9}$
UQCRC1	Down	11.27	$1.66x10^{-9}$
PSMA7	Down	11.79	$1.66x10^{-9}$
VAT1	Down	12.01	$1.66x10^{-9}$
PNP	Down	12.21	$1.66x10^{-9}$
EIF3E	Down	12.65	$1.66x10^{-9}$
SERPINA12	Down	12.74	$1.66x10^{-9}$
PSMB1	Down	12.80	$1.66x10^{-9}$
PHB1	Down	12.85	$1.66x10^{-9}$
SERPINB13	Down	13.00	$1.66x10^{-9}$
NIBAN2	Down	13.62	$1.66x10^{-9}$
ENDOU	Down	13.66	$1.66x10^{-9}$

RPS25	Down	13.94	$1.66x10^{-9}$
GLO1	Down	14.06	$1.66x10^{-9}$
ALOX12B	Down	14.08	$1.66x10^{-9}$
CAPZA2	Down	14.09	$1.66x10^{-9}$
FAM25A	Down	15.26	$1.66x10^{-9}$
SERPINC1	Down	15.31	$1.66x10^{-9}$
GAA	Down	15.50	$1.66x10^{-9}$
NCCRP1	Down	15.66	$1.66x10^{-9}$
RAN	Down	15.73	$1.66x10^{-9}$
SERPINB2	Down	16.25	$1.66x10^{-9}$
CD9	Down	16.29	$1.66x10^{-9}$
TMED10	Down	16.51	$1.66x10^{-9}$
SLC9A3R1	Down	16.94	$1.66x10^{-9}$
HSPA4	Down	17.20	$1.66x10^{-9}$
IVL	Down	17.30	$1.66x10^{-9}$
G6PD	Down	17.48	$1.66x10^{-9}$
DYNLL1	Down	18.75	$1.66x10^{-9}$
IGHV1-2	Down	19.58	$1.66x10^{-9}$
APOD	Down	20.15	$1.66x10^{-9}$
SFTPA2	Down	20.34	$1.66x10^{-9}$
SELENBP1	Down	20.42	$1.66x10^{-9}$
PSMB6	Down	20.96	$1.66x10^{-9}$
CST6	Down	21.99	$1.66x10^{-9}$
HLA-C	Down	23.73	$1.66x10^{-9}$
CAST	Down	23.93	$1.66x10^{-9}$
POF1B	Down	24.18	$1.66x10^{-9}$
SLPI	Down	24.34	$1.66x10^{-9}$
IGHV3-73	Down	25.30	$1.66x10^{-9}$
PARK7	Down	25.58	$1.66x10^{-9}$
PROM1	Down	26.92	$1.66x10^{-9}$
MMP7	Down	28.03	$1.66x10^{-9}$
UNC5C	Down	28.03	$1.66x10^{-9}$
PSMA5	Down	28.26	$1.66x10^{-9}$
SYBU	Down	30.35	$1.66x10^{-9}$
DSG3	Down	32.33	$1.66x10^{-9}$
ENPP4	Down	32.66	$1.66x10^{-9}$
KPRP	Down	36.22	$1.66x10^{-9}$
PHGDH	Down	40.03	$1.66x10^{-9}$
PRSS27	Down	41.68	$1.66x10^{-9}$
APOB	Down	42.56	$1.66x10^{-9}$
JAKMIP2	Down	43.72	$1.66x10^{-9}$
CMPK1	Down	45.15	$1.66x10^{-9}$
PLS3	Down	46.51	$1.66x10^{-9}$
HADHB	Down	51.66	$1.66x10^{-9}$
TG	Down	53.42	$1.66x10^{-9}$
ASPRV1	Down	54.25	$1.66x10^{-9}$
CD2BP2	Down	57.47	$1.66x10^{-9}$
RBP1	Down	58.12	$1.66x10^{-9}$
IGKV1-27	Down	63.88	$1.66x10^{-9}$
CUTA	Down	81.64	$1.66x10^{-9}$
ARCN1	Down	86.90	$1.66x10^{-9}$
DDOST	Down	206.72	$1.66x10^{-9}$
ZMIZ1	Down	271.71	$1.66x10^{-9}$
IGKV1D-13	Down	321.05	$1.66x10^{-9}$

Supplemental Table S2C. Submandibular DEPs

**Gene Name**[a]	**Up/Down-Regulated**[b]	**Fold Change**[c]	**p-value**[d]
IGLV1-51	Up	154.63	$3.25x10^{-8}$
PTMA	Up	109.77	$3.25x10^{-8}$
DPYSL2	Up	86.49	$3.25x10^{-8}$
BPIFB2	Up	51.32	$3.25x10^{-8}$
CHI3L2	Up	50.73	$3.25x10^{-8}$
RAB10	Up	43.55	$3.25x10^{-8}$
RAB11A	Up	43.08	$3.25x10^{-8}$
POF1B	Up	31.32	$3.51x10^{-8}$
CAST	Up	27.11	$3.25x10^{-8}$
CPA4	Up	26.79	$3.58x10^{-8}$
APOD	Up	26.37	$3.51x10^{-8}$
DDX39A	Up	24.12	$3.51x10^{-8}$
HNRNPC	Up	23.46	$3.25x10^{-8}$
IVL	Up	19.31	$3.42x10^{-8}$
A2M	Up	18.87	$3.25x10^{-8}$
PA2G4	Up	16.36	$3.25x10^{-8}$
PHB1	Up	16.31	$3.25x10^{-8}$
RCN1	Up	15.44	$3.25x10^{-8}$
CST6	Up	14.19	$3.25x10^{-8}$
CTSC	Up	13.73	$3.51x10^{-8}$
H3C12	Up	13.16	$3.25x10^{-8}$
PRDX4	Up	11.69	$3.25x10^{-8}$
UGDH	Up	11.32	$3.51x10^{-8}$
CCT3	Up	10.54	$3.25x10^{-8}$
IL6ST	Up	10.25	$3.25x10^{-8}$
CST1	Up	4.41	$1.37x10^{-2}$
TUBB2A	Up	1.16	$2.09x10^{-2}$
HNRNPU	Down	1.59	$3.48x10^{-2}$
METRNL	Down	1.71	$3.83x10^{-2}$
VCP	Down	6.79	$1.98x10^{-9}$
TOP2A	Down	6.84	$1.98x10^{-9}$
IGKV2-30	Down	7.98	$1.98x10^{-9}$
CCT4	Down	8.00	$1.98x10^{-9}$
RPL27A	Down	9.11	$1.98x10^{-9}$
LGALS1	Down	9.77	$1.98x10^{-9}$
LAMP1	Down	11.08	$1.98x10^{-9}$
PSMA7	Down	11.36	$1.98x10^{-9}$
ARPC2	Down	11.56	$1.98x10^{-9}$
ALOX12B	Down	12.09	$1.98x10^{-9}$
RPSA	Down	12.14	$1.98x10^{-9}$
RPL7	Down	13.31	$1.98x10^{-9}$
VDAC2	Down	13.63	$1.98x10^{-9}$
RPS3	Down	14.13	$1.98x10^{-9}$
ASAH1	Down	14.74	$1.98x10^{-9}$
RPS20	Down	15.05	$1.98x10^{-9}$
CRYAB	Down	16.28	$1.98x10^{-9}$
QSOX1	Down	16.39	$1.98x10^{-9}$
RPS12	Down	18.64	$1.98x10^{-9}$
IGKV1-8	Down	19.49	$1.98x10^{-9}$
ASPRV1	Down	20.97	$1.98x10^{-9}$
UBE2V1	Down	22.37	$1.98x10^{-9}$
RPS27	Down	22.76	$1.98x10^{-9}$
CCT5	Down	25.92	$1.98x10^{-9}$
MELTF	Down	28.61	$1.98x10^{-9}$
RPLP1	Down	31.09	$1.98x10^{-9}$
CRNN	Down	34.56	$1.98x10^{-9}$
CPQ	Down	36.44	$1.98x10^{-9}$
DSG3	Down	37.23	$1.98x10^{-9}$

IGHV4-28	Down	37.76	$1.98x10^{-9}$
CD2BP2	Down	38.88	$1.98x10^{-9}$
APOH	Down	39.93	$1.98x10^{-9}$
GLUD2	Down	43.00	$1.98x10^{-9}$
PARK7	Down	47.43	$1.98x10^{-9}$
SCAPER	Down	48.56	$1.98x10^{-9}$
IGKV2-28	Down	52.92	$1.98x10^{-9}$
TWF1	Down	65.88	$1.98x10^{-9}$
GLYATL3	Down	82.06	$1.98x10^{-9}$
FAM3D	Down	93.26	$1.98x10^{-9}$
GLG1	Down	124.19	$1.98x10^{-9}$
TIMP1	Down	158.63	$1.98x10^{-9}$
SPATA13	Down	189.68	$1.98x10^{-9}$
FER1L6-AS1	Down	201.84	$1.98x10^{-9}$
PC	Down	474.40	$1.98x10^{-9}$

Footnote. Differentially expressed proteins (DEPs) in xerostomic patients (XP-group) in each salivary gland. The **2A.** left parotid table includes 86 DEPs, with 47 upregulated and 39 downregulated proteins. The **2B.** right parotid table includes 112 DEPs, with 40 upregulated and 72 downregulated proteins. The **2C.** submandibular table includes 73 DEPs, with 27 upregulated and 46 downregulated proteins. Fold changes were calculated as the relative expression difference, using the formula (test - control) / control. For downregulated proteins, fold change values were expressed as the reciprocal (1/ fold change) to facilitate comparison with upregulated proteins. Statistical significance for differential expression calculated with Mann-Whitney U-test, and Benjamini-Hochberg FDR corrected.

[a]Entrez gene name
[b]Upregulation or downregulation of gene in salivary gland
[c]Relative fold change
[d]p-value (Benjamini-Hochberg corrected)
