# Supplementary material for: Salivary Proteome Profile of Xerostomic Patients Reveals Pathway Dysregulation Related to Neurodegenerative Diseases: A Pilot Study
Source: Int J Mol Sci. 2025 Jul 22;26(15):7037. doi: 10.3390/ijms26157037 (PMC12346731; doi:10.3390/ijms26157037)
Supplement: Supplementary file 1 [file ijms-26-07037-s001.zip › Xero Proteomics- Supplemental Table S3. DEPs of interest.pdf]

Supplemental Table S3: DEPs of Analytical Interest

Gene Name[a]	Salivary Gland/s[b]	Up/Down-Regulated[c]	Fold Change[d]
MUC7	LP	Up	132.36
SFN	LP	UP	63.37
LGALS7B	LP	Up	39.30
DSG3	LP;	UP;	35.90;
	RP;	Down;	32.33;
	SMSL	Down	37.23
A2M	LP;	Up;	29.03;
	SMSL	Down	18.87
HEBP2	LP	Up	28.73
GOT1	LP	Up	27.33
GLOD4	LP	Up	26.63
NPEPPS	LP	Up	24.78
RAB7A	LP	Up	22.11
RPL7	LP;	Up;	20.39;
	SMSL	Down	13.31
SBSN	LP	Up	19.54
TMED10	LP;	Up;	18.15;
	RP	Down	16.51
TYMP	LP	Up	17.45
GGCT	LP	Up	16.48
CCT5	LP;	Up;	9.01;
	SMSL	Down	25.92
*VCP**	LP;	Up;	10.49;
	RP;	Up;	10.75;
	SMSL	Down	6.79
ECH1	LP	Down	10.23
MARCHF8	LP	Down	13.14
HNRNPH3	LP	Down	14.12
*PSMA6**	LP	Down	14.64
IL6ST	LP;	Down;	14.94;
	SMSL	Up	10.25
SLC1A5	LP	Down	15.87
MUC1	LP	Down	18.10
HNRNPD	LP	Down	21.70
QSOX1	LP;	Down;	21.96;
	SMSL	Down	16.39
PAM	LP;	Down;	26.29;
	RP	Up	33.26
EGF	LP	Down	36.20
FOLR1	LP	Down	66.07
GSTA1	LP	Down	76.92
TIMP1	RP;	Up;	467.27;
	SMSL	Down	158.63
C1R	RP	Up	114.75
RAB10	RP;	Up;	28.53;
	SMSL	Up	43.55
IDUA	RP	Up	25.31
WDR1	RP	Up	25.07
CAP1	RP	Up	24.65
*TUBA4A**	RP	UP	23.67
NEU1	RP	Up	13.93
RCN1	RP;	Up;	13.56;
	SMSL	Up	15.44
RAP1B	RP	Up	13.36
ACTR3	RP	Up	9.14
UBE2N	RP	Up	8.98
CCT3	RP;	Up;	8.83;
	SMSL	Up	10.54
FASN	RP	Up	8.19
*VDAC2**	RP;	Up;	8.04;
	SMSL	Down	13.63
LGALS1	RP;	Up;	6.23;
	SMSL	Down	9.77

*PSMB5**	RP	Down	5.46
LYPD3	RP	Down	6.82
*PRNP**	RP	Down	8.92
PTBP1	RP	Down	10.04
PGM2	RP	Down	10.89
RPSA	RP;<br>SMSL	Down;<br>Down	10.96;<br>12.14
*UQCRC1**	RP	Down	11.27
*PSMA7**	RP;<br>SMSL	Down;<br>Down	11.79;<br>11.36
VAT1	RP	Down	12.01
*PSMB1**	RP	Down	12.80
PHB1	RP;<br>SMSL	Down;<br>Up	12.85;<br>16.31
SERPINB13	RP	Down	13.00
ENDOU	RP	Down	13.66
RPS25	RP	Down	13.94
GLO1	RP	Down	14.06
ALOX12B	RP;<br>SMSL	Down;<br>Down	14.08;<br>12.09
SERPINB2	RP	Down	16.25
HSPA4	RP	Down	17.20
DYNLL1	RP	Down	18.75
SELENBP1	RP	Down	20.42
*PSMB6**	RP	Down	20.96
*PARK7**	RP;<br>SMSL	Down;<br>Down	25.58;<br>47.43
UNC5C	RP	Down	28.03
*PSMA5**	RP	Down	28.26
SYBU	RP	Down	30.35
PHGDH	RP	Down	40.03
HADHB	RP	Down	51.66
RBP1	RP	Down	58.12
DDOST	RP	Down	206.72
ZMIZ1	RP	Down	271.71
DPYSL2	SMSL	Up	86.49
CHI3L2	SMSL	Up	50.73
RAB11A	SMSL	Up	43.08
HNRNPC	SMSL	Up	23.46
PRDX4	SMSL	Up	11.69
UGDH	SMSL	Up	11.32
*TUBB2A**	SMSL	Up	1.16
HNRNPU	SMSL	Down	1.59
CPQ	SMSL	Down	36.44
*GLUD2**	SMSL	Down	43.00
SCAPER	SMSL	Down	48.56
GLG1	SMSL	Down	124.19
SPATA13	SMSL	Down	189.68

Footnote. 89 differential expressed proteins (DEPs) in xerostomic patients (XP-group) were selected based on known involvement with either the oral cavity, neurological disorders, glyoxalase system, or oxidative stress. Fold changes were calculated as the relative expression difference, using the formula (test - control) / control. For downregulated proteins, fold change values were expressed as the reciprocal (1/ fold change) to facilitate comparison with upregulated proteins.

[a]Entrez gene name
[b]Salivary glands: Left Parotid (LP), Right Parotid (RP), and Submandibular (SMSL)
[c]Upregulation or downregulation of gene in salivary gland
[d]Relative fold change
Note: genes implicated in neurodegenerative diseases are depicted using asterisk (*)
