# Supplementary material for: Salivary Proteome Profile of Xerostomic Patients Reveals Pathway Dysregulation Related to Neurodegenerative Diseases: A Pilot Study
Source: Int J Mol Sci. 2025 Jul 22;26(15):7037. doi: 10.3390/ijms26157037 (PMC12346731; doi:10.3390/ijms26157037)
Supplement: Supplementary file 1 [file ijms-26-07037-s001.zip › Xero Proteomics- Supplemental Table S4. Pathways Table.pdf]

Supplemental Table S4: KEGG Pathway Enrichment Gene Sets

Supplemental Table S4A. Right Parotid KEGG Pathways

FDR and Gene Count Identifier[a]	KEGG Pathway[b]	HAS ID[c]	Count in Network[d]	Pathway FDR[e]	Gene set[f]	Up/Down-Regulated[h]	p-value[j]
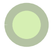	**Proteasome**	hsa03050	5 / 43	1.80×10^{-3}	***PSMA5****	Down	1.66×10^{-9}
					PSMA7	Down	1.66×10^{-9}
					PSMB1	Down	1.66×10^{-9}
					PSMB5	Down	1.66×10^{-9}
					PSMB6	Down	1.66×10^{-9}
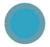	**Spinocerebellar Ataxia**	hsa05017	6 / 135	8.90×10^{-3}	***PSMA5***	Down	1.66×10^{-9}
					PSMA7	Down	1.66×10^{-9}
					PSMB1	Down	1.66×10^{-9}
					PSMB5	Down	1.66×10^{-9}
					PSMB6	Down	1.66×10^{-9}
					VDAC2	Up	2.47×10^{-8}
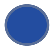	**Complement and coagulation cascades**	hsa04610	4 / 82	4.06×10^{-2}	*CFB*	Up	2.47×10^{-8}
					C1R	Up	2.47×10^{-8}
					SERPINB2	Down	1.66×10^{-9}
					SERPINC1	Down	1.66×10^{-9}
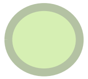	**Parkinson disease**	hsa05012	9 / 236	1.80×10^{-3}	*PARK7*	Down	1.66×10^{-9}
					PSMA5	Down	1.66×10^{-9}
					PSMA7	Down	1.66×10^{-9}
					PSMB1	Down	1.66×10^{-9}
					PSMB5	Down	1.66×10^{-9}
					PSMB6	Down	1.66×10^{-9}
					TUBA4A	Up	2.47×10^{-8}
					UQCRC1	Down	1.66×10^{-9}
					VDAC2	Up	2.47×10^{-8}
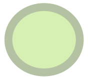	**Prion disease**	hsa05020	9 / 263	1.80×10^{-3}	*PRNP*	Down	1.66×10^{-9}
					PSMA5	Down	1.66×10^{-9}
					PSMA7	Down	1.66×10^{-9}
					PSMB1	Down	1.66×10^{-9}
					PSMB5	Down	1.66×10^{-9}
					PSMB6	Down	1.66×10^{-9}
					TUBA4A	Up	2.47×10^{-8}
					UQCRC1	Down	1.66×10^{-9}
					VDAC2	Up	2.47×10^{-8}

	**Huntington disease**	hsa05016	8 / 295	$1.42x10^{-2}$	***PSMA5***	Down	$1.66x10^{-9}$
					PSMA7	Down	$1.66x10^{-9}$
					PSMB1	Down	$1.66x10^{-9}$
					PSMB5	Down	$1.66x10^{-9}$
					PSMB6	Down	$1.66x10^{-9}$
					TUBA4A	Up	$2.47x10^{-8}$
					UQCRC1	Down	$1.66x10^{-9}$
					VDAC2	Up	$2.47x10^{-8}$
	**Alzheimer disease**	hsa05010	8 / 354	$3.59x10^{-2}$	***PSMA5***	Down	$1.66x10^{-9}$
					PSMA7	Down	$1.66x10^{-9}$
					PSMB1	Down	$1.66x10^{-9}$
					PSMB5	Down	$1.66x10^{-9}$
					PSMB6	Down	$1.66x10^{-9}$
					TUBA4A	Up	$2.47x10^{-8}$
					UQCRC1	Down	$1.66x10^{-9}$
					VDAC2	Up	$2.47x10^{-8}$
	**Ribosome**	hsa03010	5 / 131	$3.59x10^{-2}$	*RPLP0*	Up	$2.47x10^{-8}$
					RPLP1	Up	$2.51x10^{-8}$
					RPL31	Up	$2.47x10^{-8}$
					RPSA	Down	$1.66x10^{-9}$
					RPS25	Down	$1.66x10^{-9}$
	**Phagosome**	hsa04145	5 / 141	$4.06x10^{-2}$	*C1R*	Up	$2.47x10^{-8}$
					HLA-A	Up	$2.47x10^{-8}$
					HLA-C	Down	$1.66x10^{-9}$
					SFTPA2	Down	$1.66x10^{-9}$
					TUBA4A	Up	$2.47x10^{-8}$
	**Amyotrophic lateral sclerosis**	hsa05014	8 / 350	$3.59x10^{-2}$	***PSMA5***	Down	$1.66x10^{-9}$
					PSMA7	Down	$1.66x10^{-9}$
					PSMB1	Down	$1.66x10^{-9}$
					PSMB5	Down	$1.66x10^{-9}$
					PSMB6	Down	$1.66x10^{-9}$
					TUBA4A	Up	$2.47x10^{-8}$
					UQCRC1	Down	$1.66x10^{-9}$
					VCP	Up	$2.47x10^{-8}$
	**Metabolic pathways**	hsa01100	17/1435	$4.87x10^{-2}$	*ALOX12B*	Down	$1.66x10^{-9}$
					ANPEP	Up	$2.47x10^{-8}$
					DDOST	Down	$1.66x10^{-9}$
					ENPP4	Down	$2.48x10^{-9}$
					FASN	Up	$2.47x10^{-8}$
					G6PD	Down	$1.66x10^{-9}$
					GAA	Down	$1.66x10^{-9}$
					GLO1	Down	$1.66x10^{-9}$

	HADHB	Down	$1.66x10^{-9}$
	HEXA	Up	$2.47x10^{-8}$
	IDUA	Up	$2.47x10^{-8}$
	NEU1	Up	$2.47x10^{-8}$
	PGM2	Down	$1.66x10^{-9}$
	PHGDH	Down	$1.66x10^{-9}$
	PNP	Up	$2.89x10^{-8}$
	SELENBP1	Down	$1.66x10^{-9}$
	UQCRC1	Down	$1.66x10^{-9}$

Supplemental Table S4B. KEGG Pathways from DEPs of Analytical Interest

FDR and Gene Count Identifier[a]	KEGG Pathway[b]	HSA ID[c]	Count in Network[d]	FDR p-value[e]	Gene set[f]	Salivary Gland/s[g]	Up/Down-Regulated[h]	p-value[j]
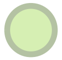	**Proteasome**	hsa03050	6 / 43	$9.36x10^{-6}$	***PSMA5****	RP	Down	$1.66x10^{-9}$
					PSMA6	LP	Down	$1.66x10^{-9}$
					PSMA7	RP;	Down;	$1.66x10^{-9}$
						SMSL	Down	$1.98x10^{-9}$
					PSMB1	RP	Down	$1.66x10^{-9}$
					PSMB5	RP	Down	$1.66x10^{-9}$
					PSMB6	RP	Down	$1.66x10^{-9}$
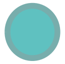	**Spinocerebellar Ataxia**	hsa05017	7 / 135	$2.40x10^{-4}$	***PSMA5***	RP	Down	$1.66x10^{-9}$
					PSMA6	LP	Down	$1.66x10^{-9}$
					PSMA7	RP;	Down;	$1.66x10^{-9}$
						SMSL	Down	$1.98x10^{-9}$
					PSMB1	RP	Down	$1.66x10^{-9}$
					PSMB5	RP	Down	$1.66x10^{-9}$
					PSMB6	RP	Down	$1.66x10^{-9}$
					VDAC2	RP;	Up;	$2.47x10^{-8}$
						SMSL	Down	$1.98x10^{-9}$
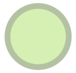	**Parkinson disease**	hsa05012	11 / 236	$4.78x10^{-6}$	*PARK7*	RP;	Down;	$1.66x10^{-9}$
						SMSL	Down	$1.98x10^{-9}$
					PSMA5	RP	Down	$1.66x10^{-9}$
					PSMA6	LP	Down	$1.66x10^{-9}$
					PSMA7	RP;	Down;	$1.66x10^{-9}$
						SMSL	Down	$1.98x10^{-9}$
					PSMB1	RP	Down	$1.66x10^{-9}$
					PSMB5	RP	Down	$1.66x10^{-9}$
					PSMB6	RP	Down	$1.66x10^{-9}$
					TUBA4A	RP	Up	$2.47x10^{-8}$
					TUBB2A	SMSL	Up	$2.09x10^{-2}$
					UQCRC1	RP	Down	$1.66x10^{-9}$
					VDAC2	RP;	Up;	$2.47x10^{-8}$
						SMSL	Down	$1.98x10^{-9}$
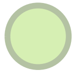	**Prion disease**	hsa05020	11 / 263	$6.93x10^{-6}$	*PRNP*	RP	Down	$1.66x10^{-9}$
					PSMA5	RP	Down	$1.66x10^{-9}$
					PSMA6	LP	Down	$1.66x10^{-9}$
					PSMA7	RP;	Down;	$1.66x10^{-9}$
						SMSL	Down	$1.98x10^{-9}$
					PSMB1	RP	Down	$1.66x10^{-9}$
					PSMB5	RP	Down	$1.66x10^{-9}$
					PSMB6	RP	Down	$1.66x10^{-9}$

				TUBA4A	RP	Up	2.47×10^{-8}
				TUBB2A	SMSL	Up	2.09×10^{-2}
				UQCRC1	RP	Down	1.66×10^{-9}
				VDAC2	RP;	Up;	2.47×10^{-8}
					SMSL	Down	1.98×10^{-9}
**Huntington disease**	hsa05016	10 / 295	9.52×10^{-5}	***PSMA5***	RP	Down	1.66×10^{-9}
				PSMA6	LP	Down	1.66×10^{-9}
				PSMA7	RP;	Down;	1.66×10^{-9}
					SMSL	Down	1.98×10^{-9}
				PSMB1	RP	Down	1.66×10^{-9}
				PSMB5	RP	Down	1.66×10^{-9}
				PSMB6	RP	Down	1.66×10^{-9}
				TUBA4A	RP	Up	2.47×10^{-8}
				TUBB2A	SMSL	Up	2.09×10^{-2}
				UQCRC1	RP	Down	1.66×10^{-9}
				VDAC2	RP;	Up;	2.47×10^{-8}
					SMSL	Down	1.98×10^{-9}
**Amyotrophic lateral sclerosis**	hsa05014	10 / 350	2.80×10^{-4}	***PSMA5***	RP	Down	1.66×10^{-9}
				PSMA6	LP	Down	1.66×10^{-9}
				PSMA7	RP;	Down;	1.66×10^{-9}
					SMSL	Down	1.98×10^{-9}
				PSMB1	RP	Down	1.66×10^{-9}
				PSMB5	RP	Down	1.66×10^{-9}
				PSMB6	RP	Down	1.66×10^{-9}
				TUBA4A	RP	Up	2.47×10^{-8}
				TUBB2A	SMSL	Up	2.09×10^{-2}
				UQCRC1	RP	Down	1.66×10^{-9}
				VCP	LP;	Up;	2.89×10^{-8}
					RP	Up;	2.47×10^{-8}
					SMSL	Down	1.98×10^{-9}
**Alzheimer disease**	hsa05010	10 / 354	2.80×10^{-4}	***PSMA5***	RP	Down	1.66×10^{-9}
				PSMA6	LP	Down	1.66×10^{-9}
				PSMA7	RP;	Down;	1.66×10^{-9}
					SMSL	Down	1.98×10^{-9}
				PSMB1	RP	Down	1.66×10^{-9}
				PSMB5	RP	Down	1.66×10^{-9}
				PSMB6	RP	Down	1.66×10^{-9}
				TUBA4A	RP	Up	2.47×10^{-8}
				TUBB2A	SMSL	Up	2.09×10^{-2}
				UQCRC1	RP	Down	1.66×10^{-9}
				VDAC2	RP;	Up;	2.47×10^{-8}
					SMSL	Down	1.98×10^{-9}

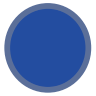	**Metabolic pathways**	hsa01100	17 / 1435	$9.50x10^{-3}$	*ALOX12B*	RP; SMSL	Down; Down	$1.66x10^{-9}$ $1.98x10^{-9}$
					DDOST	RP	Down	$1.66x10^{-9}$
					FASN	RP	Up	$2.47x10^{-8}$
					GSTA1	LP	Down	$2.48x10^{-9}$
					HADHB	RP	Down	$1.66x10^{-9}$
					IDUA	RP	Up	$2.47x10^{-8}$
					GGCT	LP	Up	$2.89x10^{-8}$
					GLO1	RP	Down	$1.66x10^{-9}$
					GLUD2	SMSL	Down	$1.98x10^{-9}$
					GOT1	LP	Up	$3.15x10^{-8}$
					NEU1	RP	Up	$2.47x10^{-8}$
					PGM2	RP	Down	$1.66x10^{-9}$
					PHGDH	RP	Down	$1.66x10^{-9}$
					SELENBP1	RP	Down	$1.66x10^{-9}$
					TYMP	LP	Up	$2.89x10^{-8}$
					UGDH	SMSL	Up	$3.51x10^{-8}$
					UQCRC1	RP	Down	$1.66x10^{-9}$

Footnote. KEGG enrichment gene sets for each pathway from differential expressed proteins (DEPs) in the **4A.** right parotid and all **4B.** DEPs of analytical interest from each salivary gland selected based on known involvement with either the oral cavity, neurological disorders, glyoxalase system, or oxidative stress.

For Supplemental Table S4A and S4B:

[a]Pathway FDR Identifier (light green colors for significant low FDR values compared to darker blue shading for high FDR values) and Gene Counts represented by the size of the circle
[b]KEGG Pathway
[c]HSA ID for KEGG Pathway
[d]Observed gene count in KEGG network
[e]Pathway FDR p-value (Benjamini – Hochberg corrected)
[f]Gene Set (Entrez gene name)
[g] Salivary glands: Left Parotid (LP), Right Parotid (RP), and Submandibular (SMSL)
[h]Upregulation or downregulation of gene in the right parotid or [h]Left Parotid (LP), Right Parotid (RP), and Submandibular (SMSL)
[i]FDR p-value (Benjamini-Hochberg corrected)
*Note: Proteasome Core Complex (PSM) proteins in common between neurodegenerative disease pathways are highlighted in **bold**
